# Amelogenesis Imperfecta: Full Mouth Rehabilitation in Deciduous Dentition

**DOI:** 10.5005/jp-journals-10005-1105

**Published:** 2010-04-15

**Authors:** Pinky, Satyajith Naik, Shashikiran ND

**Affiliations:** 1 Senior Lecturer, Department of Pediatric Dentistry, Peoples College of Dental Sciences and Research Center Bhopal, Madhya Pradesh, India; 2 Professor, Department of Pediatric Dentistry, College of Dental Sciences, Davangere, Karnataka, India; 3 Dean, Peoples College of Dental Sciences and Research Center, Bhopal, Madhya Pradesh, India

**Keywords:** Deciduous dentition, Amelogenesis imperfecta, Stainless steel crowns, Indirect composite veneers.

## Abstract

This clinical report describes the oral rehabilitation of a very young child diagnosed with hypoplastic amelogenesis imperfecta. The specific treatment objectives being adequate patient management, eliminate tooth sensitivity while enhancing esthetics, masticatory function and improved self confidence. The treatment included full mouth rehabilitation with stainless steel crowns on posterior teeth and indirect composite veneers on anterior teeth.

## INTRODUCTION

Genetic defects of enamel are the most frequent anomalies of dental hard tissues.^1^ It has been described as a complex group of inherited conditions that disturbs the developing enamel structure and exists independent of any related systemic disorder.^2,3^ The condition affects both primary and permanent teeth and causes them to be unusually small, discolored, pitted, grooved and prone to rapid wear and breakage.^4^ Incidence varies from 1:718 to 1:14,000 depending on the population studied.^5^ The condition has been divided into four main types (hypoplastic, hypomaturative, hypocalcification and hypomaturation―hypoplastic with taurodontism) with 14 subtypes based on clinical, radio-graphic and genetic factors.^5^ The hypoplastic is characterized by defects in enamel matrix formation with pitted and grooved enamel. Hypocalcified type is due to defects in calcification of enamel, enamel is formed to normal thickness but weak, opaque/chalky in appearance with rapid wear. Hypomaturative type is characterized by enamel of normal thickness and matted in appearance, enamel is softer and vulnerable to attrition, but not as severely as hypocalcified type and hypoplastic- hypomaturative with taurodontism is characterized by mixed hypomaturation/ hypoplastic appearance, and taurodontism is a common factor.^6^ According to literature amelogenesis imperfecta (AI) patients regardless of subtypes have similar oral complications: teeth sensitivity, poor esthetics, decreased vertical dimension and reduced self confidence when anterior teeth are affected.^2,7^ From the point of esthetic rehabilitation, two problems are encountered in primary dentition; small teeth dimensions, thus a high risk of pulp injury during preparation and immense difficulty in performing tooth preparation with rotary instruments in children 3 to 5-year-old. Unlike the permanent dentition, crown restorations in primary dentition are exposed to less masticatory loading and the period of use is limited in most cases to 5 years or less. The application of total bonding and adhesive luting techniques allow clinically reliable placement of resin composite and stabilization of remaining tooth substance. The aim of this work is to present a clinical method for veneer restoration in primary teeth affected by AI that does not require previous preparation by rotary instruments and is applicable in children younger than 5-year-old.^7^

## CASE REPORT

**Fig. 1:**
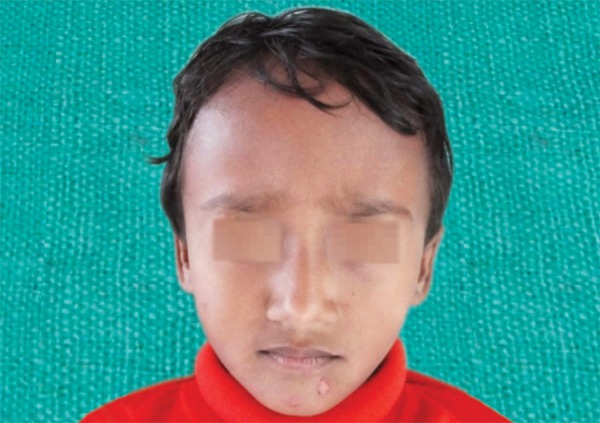
Preoperative facial photograph

**Fig. 2:**
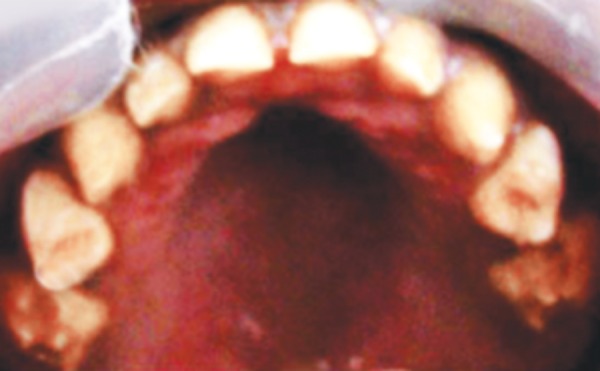
Preoperative maxillary occlusal view

**Fig. 3:**
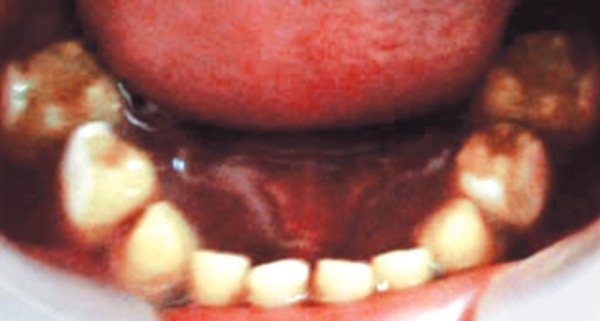
Preoperative mandibular occlusal view

**Fig. 4:**
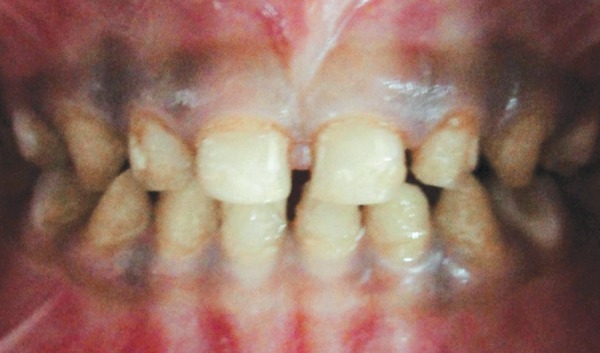
Preoperative occlusal view

**Fig. 5:**
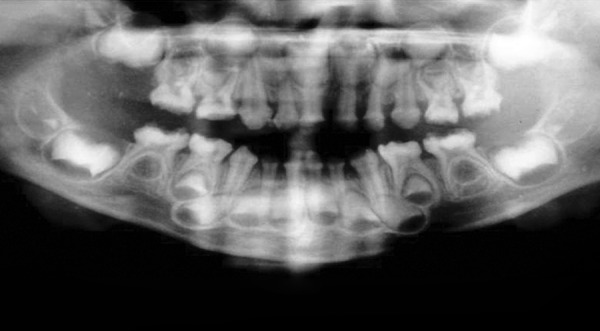
OPG showing open contacts and incompletely formed enamel affecting both deciduous and underlying permanent teeth

A 3-year-old healthy boy was referred to our clinic with a request for dental care for his esthetic problems and sensitive teeth. He expressed extreme dissatisfaction with his appearance and his father confirmed that patients social life was affected by this problem due to being teased by other children (Fig. 1). The medical history indicated no contraindications for dental treatment. A detailed extra- and intraoral clinical examination and radiographic evaluation were performed. The patient had full deciduous dentition. The enamel layer of all teeth was very thin and yellowish brown in color and cuspal structure nearly flattened in posterior region (Figs 2 and 3). The exposed dentin was dark yellow in color and hypersensitive (Fig. 4). Periapical and panoramic radiograph revealed loss of enamel, especially on occlusal surfaces of posterior teeth and proximally (Fig. 5). The molars were in mesial step molar relation and upper anteriors with an overjet and overbite of about 2 mm. Oral hygiene was not satisfactory with evidence of gingivitis.

**Fig. 6:**
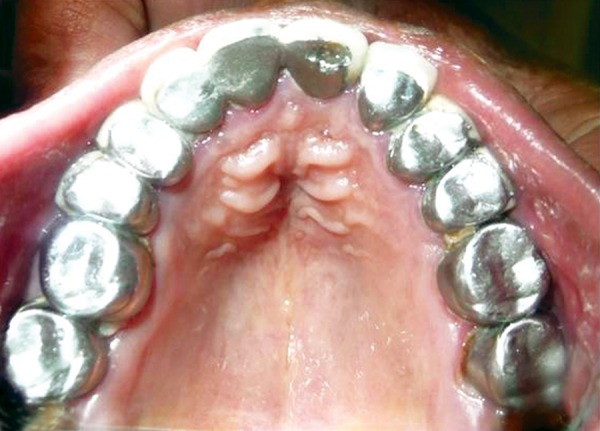
Maxillary occlusal view of mother’s dentition with prosthetic rehabilitation

**Fig. 7:**
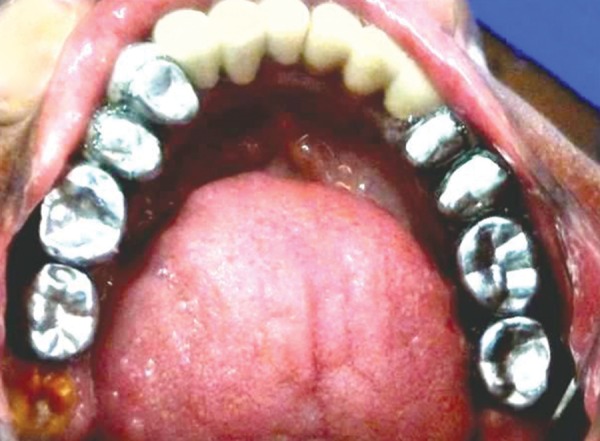
Mandibular occlusal view of mother’s dentition with prosthetic rehabilitation and partially erupted hypoplastic 38

There was a history of consanguineous marriage. They were further questioned about the presence of similar abnormalities in the family, including grandparents. Patient’s mother and his sister gave a history of similar condition. Mother had undergone full mouth rehabilitation for esthetic and functional purpose (Fig. 6). The same was confirmed with her nonrestored partially erupted third molars which were indicated for extraction (Fig. 7). Following extraction, the specimen was sent for a histopathologic examination, and the clinical diagnosis was confirmed to be of hypoplastic type of amelogenesis imperfecta.

## TREATMENT

The patient was informed for the diagnosis, and all the treatment modalities of full mouth rehabilitation were explained to patient and the parent. The patient was placed on intensive oral hygiene program, including scaling, and after 2 weeks the level of oral hygiene maintained by the patient was acceptable with improvement in soft tissues.

All posterior molars were restored with 3M S.S crowns and indirect veneers were fabricated for maxillary and mandibular anteriors due to uncooperative nature of the young pediatric patient.

**Fig. 8:**
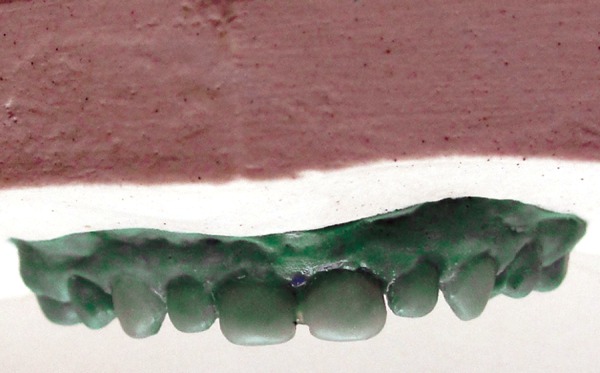
Indirectly fabricated composite veneers

**Fig. 9:**
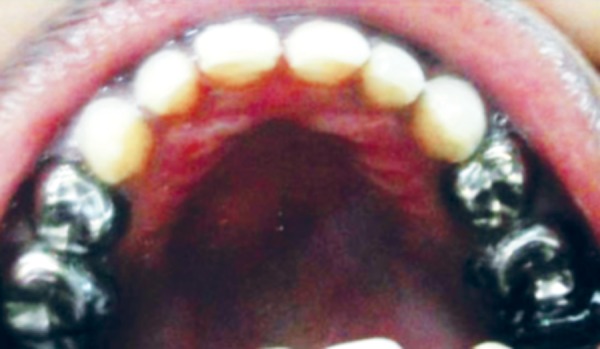
Postoperative maxillary occlusal view

Fabrication of resin composite veneers:

- Alginate impressions were made and poured with dental stone to obtain master casts.
- Following which separating media was applied.
- Extraoral veneers were fabricated with light cure composite resin (A1 shade, 3 MESPE). The veneers were designed with a large coronal part covering the exposed dentin and a thin gingival part covering the remaining enamel with veneer margins ending 0.5 mm from attached gingival/equigingival (Fig. 8).

### Luting the Restorations

**Fig. 10:**
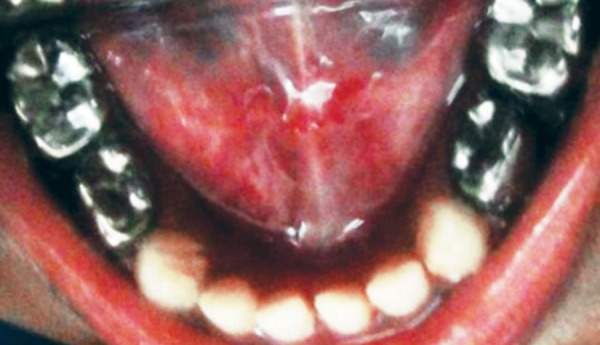
Postoperative mandibular occlusal view

**Fig. 11:**
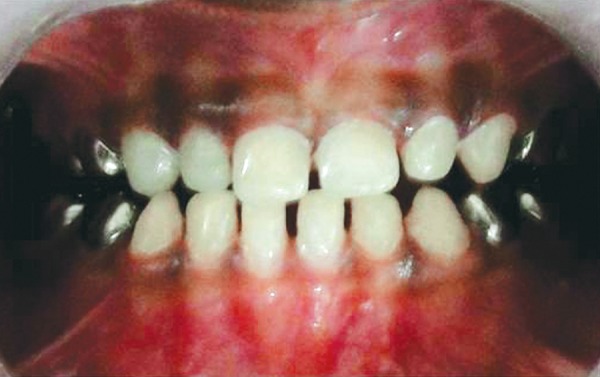
Postoperative occlusal view

**Fig. 12:**
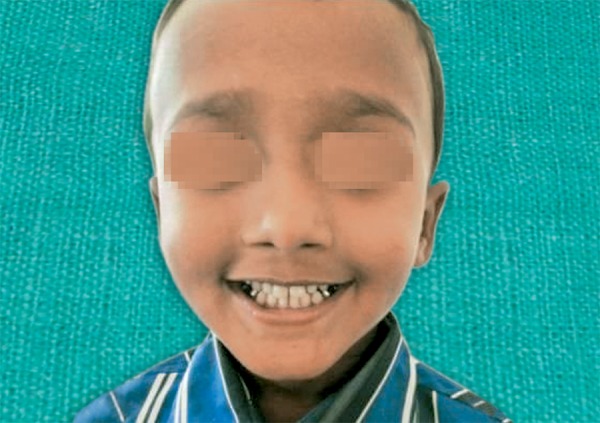
Postoperative facial photograph showing patient with improved confidence

After the teeth are cleaned with cotton pellet, they were isolated with cotton rolls and high volume suction. All teeth were acid-etched for 30 seconds with 37% H_3_PO_4_ (Total etch, Vivadent) thoroughly washed and dried with cotton pellets. Single bond adhesive system (3M/ESPE) was applied for 20 seconds on the enamel and dentin and light cured for 10 seconds. The composite resin veneers were then covered with flowable composite and positioned onto teeth. The excess composite being removed with a scaler, followed by gentle air stream and light cured twice as long (80 second) as recommended by the manufacturer. After placement of all restorations, the occlusion was checked and adjusted to correct vertical dimension (Figs 9 to 11). After the restorative procedure, the patients dentin hypersensitivity disappeared completely and functional chewing was established. The patient was recalled 6 and 12 months after the treatment, wherein psychology of patient was found to have improved greatly due to esthetic rehabilitation (Fig. 12).

## DISCUSSION

A treatment plan for cases of AI is related to many factors: Age and the socioeconomic status of the patient, the type and severity of disorder and the intraoral situation at the time treatment was planned.^8,9^

Historically, some patients with AI have been treated with multiple extractions followed by construction of complete dentures. These options are psychologically harsh, especially in adolescents.^10^ Because of the advances in esthetic dentistry, especially in bonding to dentin; today it is possible to restore function and esthetics to an acceptable level and for a long time, but an extension of a direct restoration onto all surfaces of tooth crown often results in an incorrect or at least not optimal anatomic form and insufficient adaptation. Therefore, teeth severely affected by AI have been restored with crowns luted after tooth preparation by rotary instruments.

However, the use of rotary instruments in children younger than 4 years old is difficult and the danger of injury of the pulp is obvious. Thus, we have followed the concept of laboratory fabricated resin composite veneers luted on totally etched and bonded teeth affected with AI.^11-13^

It should be noted that bonding of resin composite to the residual enamel of teeth affected by AI is often problematic, especially in individual cases with poorly mineralized friable enamel. However, the SEM investigation revealed that the rough dentinal surface and the enamel margins supply a multitude of retentive areas, increasing the restorations inter-facial bonding and stability. Moreover, sealing of the dentinal tubules remedied the dentinal hypersensitivity. In addition, adhesive luting of the restoration to the remaining enamel supplies a high quantity of microretentions enabling very stable anchorage of the restorations. The esthetic appearance of the restoration can be additionally improved by a layer of opaquer, particularly in teeth with severe discoloration.^14^

## CONCLUSION

This case report describes an easy to follow, noninvasive approach for restoration of clinical crowns of primary teeth with complete or partial loss of enamel. The restorative technique using indirect crowns and veneers requires no tooth preparation with rotary instruments and can be used in children as young as 3 to 5-year-old. Extraoral fabrication of restoration makes treatment shorter and more reliable.
